# Design and Evaluation of a New Resin-Filled GFRP Pipe Connection System for Butt Splicing of FRP Bars

**DOI:** 10.3390/ma14010161

**Published:** 2020-12-31

**Authors:** Hui Huang, Jie Lian, Jiaxing Li, Bin Jia, Dong Meng, Zhizhong Wu

**Affiliations:** 1 School of Civil Engineering and Architecture, Southwest University of Science and Technology, Mianyang 621010, China; lijiaxing950828@163.com (J.L.); jiabin@swust.edu.cn (B.J.); m17771432470@163.com (D.M.); WZZ336699@139.com (Z.W.); 2 Central Research Institute of Building and Construction Co, Beijing 100088, China; lianjiezy@163.com

**Keywords:** FRP bars, GFRP pipe, connection system, interface, tensile capacity, failure mode

## Abstract

Fiber-reinforced polymer (FRP) bars are one of the promising alternatives for steel bars used in concrete structures under corrosion or non-magnetic environments due to the unique physical properties of FRP materials. When compared with steel bars, FRP bars are difficult to be spliced in field application due to their anisotropy and low shear and compressive strengths. In view of this, the paper presents a new non-metallic connection system (i.e., resin-filled glass fiber-reinforced polymer (GFRP) pipe connection system) for the butt splicing of FRP bars. With the proposed connection system and a simplified trilinear interfacial bond-slip model, a set of design formulas were derived based on the requirement that the proposed connection system should provide a load transfer capacity beyond the tensile capacity of the spliced FRP bars (i.e., to fulfill the high tensile strength of FRP materials). Besides, considering the fabrication error-induced load transfer capacity reduction of the connection system in field application, a correction factor was introduced in the paper to compensate for the reduced load transfer capacity by increasing the FRP bar anchorage length. At last, to estimate the effectiveness of the proposed connection system and the derived design formulas, nine specimens were fabricated with a kind of commercially available basalt fiber-reinforced polymer (BFRP) bars and the designed connection system and tested under unidirectional tension to study their tensile performance. With the comparison between the tested and theoretical results, the effectiveness of the proposed connection system and the derived design formulas are verified.

## 1. Introduction

For decades, fiber-reinforced polymer (FRP) bars have been gradually used extensively in civil engineering due to the unique merits of light weight [1], high strength [2,3], fatigue and corrosion resistance [4,5,6,7], non-magnetic [8,9,10,11], reversibility [12] and the easy incorporation with fiber sensors [13,14]. Due to the brittle property of FRP materials, FRP bars are usually fabricated with a specific length for the convenience of storage and transportation, and thus they need to be spliced in field application, especially in high-rise or long-span structures. Whereas, when compared with steel reinforcement, FRP bars are difficult to be spliced due to the anisotropy and low shear and compressive strengths of FRP materials [15,16]. Thus, the development of a reliable connection system for FRP bars is a key issue to be addressed in field application.

According to the literature research conducted by the authors, relatively few studies [17,18] are focused on the development of a connection system for the butt splicing of FRP bars. In these studies, Huang et al. [18] proposed the resin-filled round steel pipe connectors for the butt splicing basalt fiber-reinforced polymer (BFRP) bars and conducted a set of tensile tests to verify the effectiveness of the connection system with four variables. Based on the tested results, the proposed connection system was found to be able to provide a reliable connection for the BFRP bars studied in the paper. As referring to connect glass fiber-reinforced polymer (GFRP) bars with shape memory alloy (SMA) bars, Alam et al. [17] provided the adhesive-type and mechanical-type couplers and conducted a series of unidirectional tensile tests on the proposed couplers. The test results showed that adhesive-type couplers can be applied to GFRP bar connection and mechanical-type couplers should not be adopted. Since the latter causes damage to the surface of the GFRP bars, and thus induces premature failure.

Compared with the aforementioned few pieces of literature, a lot of work that can be retrieved is related to the development of systems for anchoring FRP rods/tendons [19,20,21,22,23,24,25,26,27,28,29,30,31]. Since the connection system to be developed plays a role similar to an anchorage end used to anchor two symmetrically placed FRP bars, these works can also be referenced in the paper. Considering the different load transfer mechanisms, these systems can be classified into two types, namely wedge-type [19,20,21,22,23,24,25,26,27] and bond-type anchorages [28,29,30,31].

A wedge-type anchorage usually consists of metallic or non-metallic wedges to grip the FRP rods/tendons placed in a round steel tube with the designed inner conical profile [19,20,21,22,23]. The mechanical behavior of a wedge-type anchorage is highly dependent on the interaction activated between the wedges and the FRP rods/tendons. Although the metallic wedge-type anchorages have been verified effectively in some experimental tests, the wedges tend to introduce surface damage on FRP rods/tendons that significantly reduce the tensile performance. The need to reduce metallic wedges induced damage means the wedges should be shallower and much larger than those for steel tendons, which may significantly increase the size of the anchorage end. Apart from the efforts spent on improving the geometrical configuration of the metallic wedges, other efforts, such as soft metal overlay, plastic wedges, have also been tried to improve the efficiency of the wedge-type anchorages [22]. Whereas, due to the complex configuration of wedge-type anchorages, the drawbacks are high manufacturing costs, complex assembly procedures, and long assembly time.

Compared with wedge-type anchorages, bond-type anchorages have also been testified widely for anchoring FRP rods/tendons [28,29,30,31]. A bond-type anchorage is usually fabricated with a steel housing and the grouted bonding layer. The mechanical behavior of a bond-type anchorage is highly dependent on the internal configuration of the housing, the external configuration and the anchorage length of FRP rods/tendons, the mechanical behavior of the grout material. Compared with wedge-type anchorages, bond-type anchorages can be fabricated in a relatively simple way by anchoring the FRP rods/tendons in a profiled steel sleeve to the grouted bonding agent. Whereas, due to the introduction of steel housing, the drawbacks of traditional bond-type anchorages are heavy, low-durability, easy electric and magnetic conductivity.

Although the aforementioned literature proposed some connection methods for FRP bars and revealed some mechanical performance of various wedge-type and bond-type anchorages for FRP rods/tendons, the development of a reliable non-metallic connection system for the butt splicing of FRP bars to fulfill the unique merits of FRP materials is still insufficient. In view of this, the objectives of the research are (1) to propose a non-metallic connection system (i.e., resin-filled GFRP pipe connection system) for the butt splicing of FRP bars; (2) to derive a set of formulas for designing the proposed connection system; and (3) to evaluate the effectiveness of the proposed resin-filled GFRP pipe connection system and the derived design formulas with a set of experimental tests.

## 2. The Connection System Design

### 2.1. Prototype Design

The proposed connection system should have properties similar to those of the spliced FRP bars to fulfill the functional merits. Besides, the proposed connection system should also be small-sized, low-cost, and have easy-manufacturing, and handling to facilitate the massive field application.

**Figure 1:**
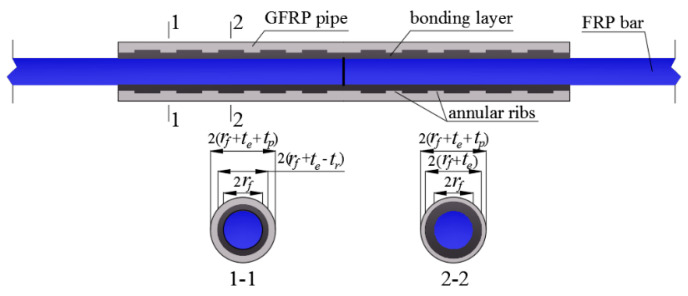
Proposed resin-filled GFRP pipe connection system for the butt splicing of FRP bars.

Based on the aforementioned functional requirements, a new resin-filled GFRP pipe connection system is proposed in the paper for the butt splicing of FRP bars. The connection system is composed of a round GFRP pipe with evenly distributed internal annular ribs and a grouted bonding layer with uneven thickness along the longitudinal direction (Figure 1).

### 2.2. Configuration Design

For the proposed resin-filled GFRP pipe connection system, many mechanical requirements need to be satisfied in field application. Among the various mechanical requirements, the quasi-static load transfer capacity is the fundamental one. Since the proposed connection system should provide a reliable load transfer capacity to the spliced FRP bars to fulfill the high strength of FRP materials (i.e., to ensure FRP bar rupture) under unidirectional tension.

**Figure 2:**
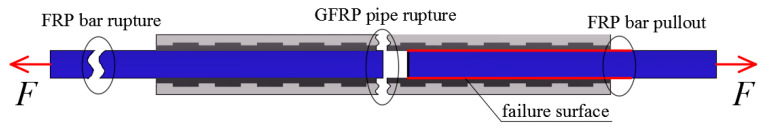
Failure modes of FRP bars spliced with the proposed connection system.

The working mechanism of the proposed connection system is to splice two symmetrically placed FRP bars and ensure the load is transferred reliably from one bar to the other bar through the bonding layer, the GFRP pipe, and the interfaces (i.e., GFRP pipe-bonding layer interface and FRP bar-bonding layer interface). With the symmetrical load transfer path, the possible failure modes of FRP bars spliced with the proposed connection system under unidirectional tension can be classified into three types, namely FRP bar rupture, GFRP pipe rupture, and FRP bar pullout, as shown in Figure 2.

In these failure modes, the desired one is FRP bar rupture which means the connection system performs well in transfer the tensile force and the high strength of FRP bars can fully be utilized. Whereas the rest two premature failures (i.e., GFRP pipe rupture and FRP bar pullout) may occur at a low load that restricts the strength development in the spliced FRP bars. Based on the above analysis and the configuration of the proposed connection system, the load transfer capacity of the connection system is highly dependent on three parameters (i.e., the net bonding layer thickness, the net GFRP pipe wall thickness, and the FRP bar anchorage length), and thus the detailed design methods for determining each of the three parameters will be presented in the following paragraphs with the consideration to ensure the connection system provides a load transfer capacity beyond the tensile capacity of the spliced FRP bars.

#### 2.2.1. The Net Bonding Layer Thickness Design

In a previous experimental study on resin-filled round steel pipe connectors for the butt splicing of BFRP bars [18], a minimum net bonding layer thickness of 3 mm was found both convenient for field operation and capable to guarantee the quality of the grouted bonding layer for the commonly used epoxy resins, and thus a net bonding layer thickness of 3 mm is adopted herein.

#### 2.2.2. The Net GFRP Pipe Wall Thickness Design

In determining the net GFRP pipe wall thickness, the following two aspects should be considered simultaneously, namely (1) the GFRP pipe thickness should be as small as possible to facilitate field application and satisfy structural requirements of concrete structures and (2) the GFRP pipe should provide a net thickness large-enough to sustain a load beyond the tensile capacity of the spliced FRP bars. In view of these, the net GFRP pipe wall thickness (*t*_p_) can be designed with the following equation under the given FRP bar radius and the bonding layer thickness.

(1) $$\sigma_{\mathrm{pu}}\pi \left[{\left(r_{f}+t_{e}+t_{p}\right)}^{2}-{\left(r_{f}+t_{e}\right)}^{2}\right]\geq \sigma_{\mathrm{fu}}\pi r_{f}^{2}$$

where σ_pu_ is the ultimate tensile strength of the GFRP pipe; σ_fu_ is the ultimate tensile strength of the FRP bars to be spliced; *r*_f_ is the FRP bar radius; *t*_e_ is the bonding layer thickness (i.e., the bonding layer thickness at the sections without annular ribs, as shown in Figure 1).

#### 2.2.3. The FRP Bar Anchorage Length Design

Compared with the net bonding layer thickness and the net GFRP pipe wall thickness, the determination of FRP bar anchorage length (i.e., the GFRP pipe length) is much more difficult, since the FRP bar anchorage length is directly related to the premature failure of FRP bar pullout. For an FRP bar pulled out from the resin-filled GFRP pipe connection system proposed in the paper, the possible failure surfaces are the FRP bar-bonding layer interface and the FRP bar-GFRP pipe wall interface. Whereas, considering the larger contact area and the designed annular ribs induced external mechanical contribution on the GFRP pipe wall-bonding layer interface, the failure surface for FRP bar pullout is more likely to be located at the FRP bar-bonding layer interface. In view of this, the following sections will be focused on in the calculation of the critical FRP bar anchorage length (i.e., the minimum FRP bar anchorage length) to ensure the interface provides a load transfer capacity equal to the tensile capacity of the spliced FRP bars.

1. Basic assumptions and equations

**Figure 3:**
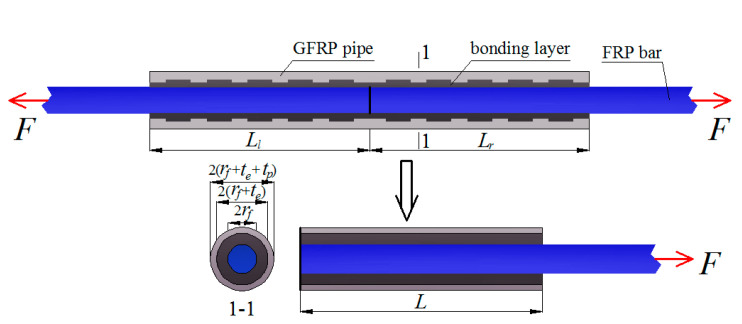
Theoretical model for FRP bars spliced with the proposed connection system.

**Figure 4:**
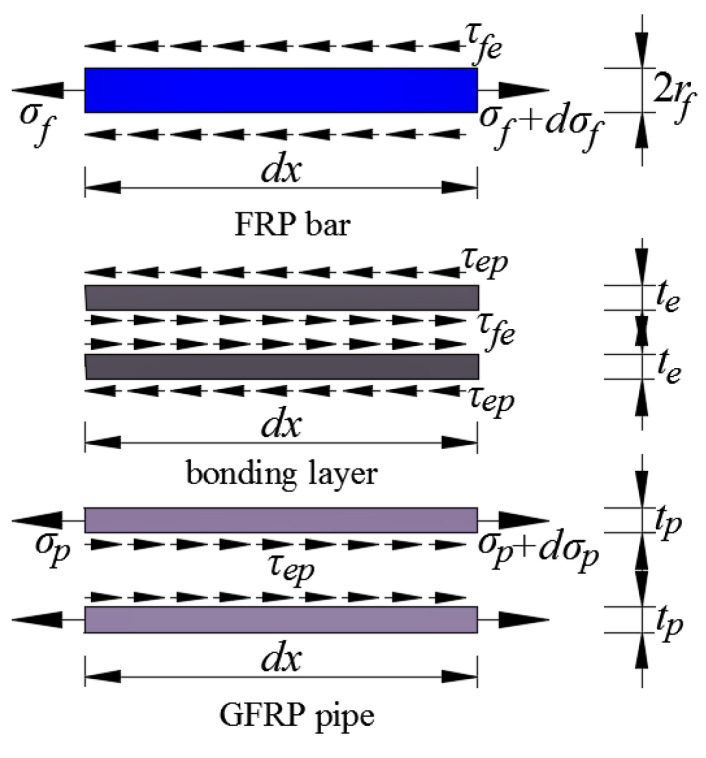
Mechanical analysis of the differential element.

In determining the critical FRP bar anchorage length, a typical resin-filled GFRP pipe connection system is shown in Figure 3, where *r*_f_, *t*_p_, *t*_e_, and *L* are the FRP bar radius, the net GFRP pipe wall thickness, the bonding layer thickness, and the critical FRP bar anchorage length to be determined, respectively. In addition, it should be mentioned that the external annular ribs are ignored in the model to simplify the theoretical derivation. To analyze the shear behavior of the FRP bar-bonding layer interface, an element is chosen along the length of the connection system for theoretical derivation (as shown in Figure 4). For the FRP bar element, according to the balance of force in the longitudinal direction, we have:

(2) $$\frac{d\sigma_{f}}{dx}=\frac{C_{f}}{A_{f}}\tau_{\mathrm{fe}}$$

where σ_f_ is the tensile stress of the FRP bar element; τ_fe_ is the shear stress on the interface between the FRP bar and the bonding layer; *A*_f_ and *C*_f_ are the cross-sectional area and the perimeter of the FRP bar, respectively. The two parameters are:

(3) $$A_{f}=\pi r_{f}^{2}$$

(4) $$C_{f}=2\pi r_{f}$$

Since FRP materials show the linear-elastic behavior under unidirectional tension, the tensile stress σ_f_ in the FRP bar element can be depicted with the tensile strain ε_f_.

(5) $$\sigma_{f}=E_{f}\varepsilon_{f}=E_{f}\frac{du_{f}}{dx}$$

where *u*_f_ is the longitudinal displacement of the FRP bar; *E*_f_ is the elastic modulus of the FRP bar.

With the combination of Equations (2) and (5), we have:

(6) $$E_{f}\frac{d^{2}u_{f}}{dx^{2}}=\frac{C_{f}}{A_{f}}\tau_{\mathrm{fe}}$$

Conducting the same reasoning again, the longitudinal displacement *u*_p_ of the GFRP pipe element is depicted as follows.

(7) $$E_{p}\frac{d^{2}u_{p}}{dx^{2}}=-\frac{C_{p}}{A_{p}}\tau_{\mathrm{ep}}$$

where *E*_p_ is the elastic modulus of the GFRP pipe; *C*_p_ is the inner perimeter of the GFRP pipe; *A*_p_ is the cross-sectional area of the GFRP pipe; τ_ep_ is the shear stress on the interface between the GFRP pipe and bonding layer. The two parameters *C*_p_ and *A*_p_ are equal to:

(8) $$C_{p}=2\pi \left(r_{f}+t_{e}\right)$$

(9) $$A_{p}=\pi [{\left(r_{f}+t_{e}+t_{p}\right)}^{2}-\left(r_{f}+t_{e}{)}^{2}\right]$$

According to the findings of Zheng et al. [26], Benmokrane et al. [32], and Wu et al. [33], the load sustained by the bonding layer can be regarded as simply shear. Thus, the shear-induced stress τ in the bonding layer is:

(10) $$\tau =\frac{r_{f}}{r}\tau_{\mathrm{fe}}$$

where *r* is the distance from the given point in the bonding layer to the center of the FRP bar. According to the sectional configuration of the proposed connection system, the *r* varies from *r*_f_ to *r*_f_ + *t*_e_.

If *r* equals to *r*_f_ + *t*_e_, the interfacial shear stress τ_ep_ on the GFRP pipe wall-bonding layer, the interface can be depicted as:

(11) $$\tau_{\mathrm{ep}}=\frac{C_{f}}{C_{p}}\tau_{\mathrm{fe}}$$

With the combination of Equations (7) and (11), we have:

(12) $$E_{p}\frac{d^{2}u_{p}}{dx^{2}}=-\frac{C_{f}}{A_{p}}\tau_{\mathrm{fe}}$$

Considering Hook’s law in shear, the relationship between the shear stress *τ* and the displacement *u*_e_(*r*) of the bonding layer element can be expressed as:

(13) $$\tau =-G_{e}\frac{du_{e}\left(r\right)}{dr}$$

where *G*_e_ is the shear modulus of the bonding material.

With the combination of Equations (10) and (13), the following equation can be derived:

(14) $$du_{e}\left(r\right)=-\frac{\tau_{\mathrm{fe}}r_{f}}{G_{e}}\frac{dr}{r}$$

By integrating Equation (14) with the variable *r* from *r*_f_ to *r*_f_ + *t*_e_, the shear stress-induced shear deformation of the bonding layer can be obtained as:

(15) $$u_{e}\left(r_{f}+t_{e}\right)-u_{e}\left(r_{f}\right)=-\frac{\tau_{\mathrm{fe}}r_{f}}{G_{e}}ln(\frac{C_{p}}{C_{f}})$$

If the bond on the interface between the GFRP pipe wall and the bonding layer is assumed to be perfect, the displacement of the GFRP pipe is equal to the displacement of the bonding layer at *r*_f_ + *t*_e_, that is:

(16) $$u_{p}=u_{e}\left(r_{f}+t_{e}\right)$$

**Figure 5:**
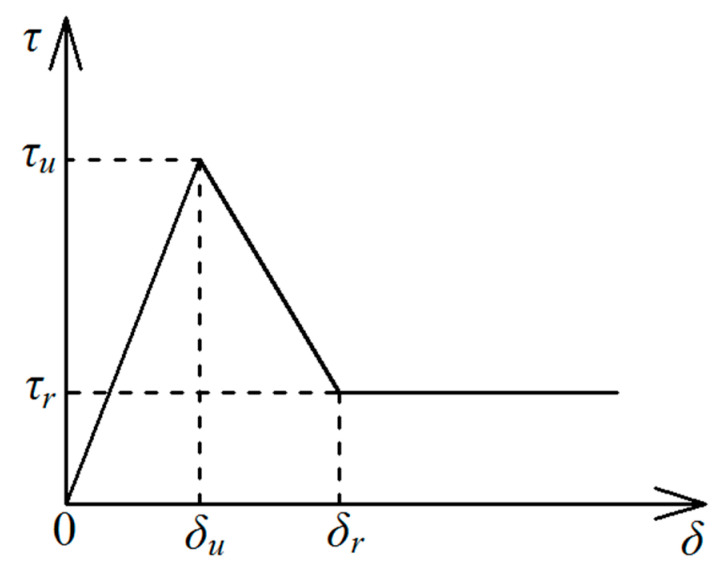
The trilinear bond-slip curve.

With the research results of some pioneer works [26,32,33], the interfacial shear behavior of the FRP bar-bonding layer interface can be described with a simplified trilinear bond-slip curve, as seen in Figure 5.

The trilinear bond-slip curve is described as:

(17) $$\tau_{\mathrm{fe}}=\{\begin{array}{cc} \frac{\tau_{u}}{\delta_{u}}\delta & 0\leq \delta \leq \delta_{u}\\ \frac{\delta_{r}\tau_{u}-\delta_{u}\tau_{r}}{\delta_{r}-\delta_{u}}-\frac{\tau_{u}-\tau_{r}}{\delta_{r}-\delta_{u}}\delta & \delta_{u}\leq \delta \leq \delta_{r}\\ \tau_{r} & \delta \geq \delta_{u} \end{array}$$

where τ_u_ is the peak shear stress; δ_u_ is the relative slip at τ_u_; τ_r_ is the residual frictional stress; δ_r_ is the relative slip at the end of the softening part (as seen in Figure 5).

Considering the deformation compatibility, the relative slip δ between the FRP bar element and the bonding layer element is:

(18) $$\delta =u_{f}-u_{e}\left(r_{f}\right)$$

By taking Equations (16) and (18) into Equation (15), the following equation can be obtained:

(19) $$u_{f}-u_{p}-\delta =\frac{\tau_{\mathrm{fe}}r_{f}}{G_{e}}\ln(\frac{C_{p}}{C_{f}})$$

2. Theoretical solution

As seen in Figure 5, the interfacial bond-slip curve is composed of three parts: elastic part, softening part, and frictional part. Therefore, in determining the critical FRP bar anchorage length, the theoretical solution for each part should be conducted.

(1) Elastic part

In the elastic part, with the combination of Equations (17) and (19), the relative displacement between the FRP bar and the GFRP pipe is:

(20) $$u_{f}^{e}-u_{p}^{e}=\left[\frac{\delta_{u}}{\tau_{u}}+\frac{r_{f}}{G_{e}}\ln(\frac{C_{p}}{C_{f}}\right)]\tau_{\mathrm{fe}}^{e}$$

In Equation (20), quantities with the superscript *e* are all defined in the elastic part.

If *k*_1_, *k*_2_, and *r*_1_ are depicted with the following expressions:

(21) $$k_{1}=\frac{C_{f}}{E_{f}A_{f}\left[\frac{\delta_{u}}{\tau_{u}}+\frac{r_{f}}{G_{e}}\ln(\frac{C_{p}}{C_{f}}\right)]}$$

(22) $$k_{2}=\frac{C_{f}}{E_{p}A_{p}\left[\frac{\delta_{u}}{\tau_{u}}+\frac{r_{f}}{G_{e}}\ln(\frac{C_{p}}{C_{f}}\right)]}$$

(23) $$r_{1}=\sqrt{k_{1}+k_{2}}$$

Then, Equations (6) and (12) can be simplified as:

(24) $$\{\begin{array}{c} \frac{d^{2}u_{f}^{e}}{dx^{2}}-k_{1}u_{f}^{e}+k_{1}u_{p}^{e}=0\\ \frac{d^{2}u_{p}^{e}}{dx^{2}}+k_{2}u_{f}^{e}-k_{2}u_{p}^{e}=0 \end{array}$$

With the method for ordinary differential equation solution, the solved result of Equation (24) is:

(25) $$\left\{\begin{array}{c} u_{f}^{e}\\ u_{p}^{e} \end{array}\right\}=\eta \exp(\mu x)$$

where η is a vector. With the combination of Equations (23) and (24) and considering the non-zero solution of Equation (24), the following characteristic equation can be derived:

(26) $$\left|\begin{array}{c} \mu^{2}-k_{1}\;\;\;\;\;k_{1}\\ k_{2}\;\;\;\;\;\mu^{2}-k_{2} \end{array}\right|=0$$

By solving Equation (26), the values of μ are:

(27) $$\mu_{1}=\mu_{2}=0;\;\mu_{3}=-\mu_{4}=r_{1}$$

The calculated characteristic vectors are expressed as follows:

(28) $$\eta_{1}=\eta_{2}=\left\{\begin{array}{c} 1\\ 1 \end{array}\right\};\eta_{3}=\eta_{4}=\left\{\begin{array}{c} k_{1}\\ -k_{2} \end{array}\right\}$$

With the characteristic vectors, the solutions of Equation (24) are:

(29) $$\{\begin{array}{c} u_{f}^{e}=a_{1}+a_{2}x+a_{3}k_{1}\cosh r_{1}x+a_{4}k_{1}\sinh r_{1}x\\ u_{p}^{e}=a_{1}+a_{2}x-a_{3}k_{2}\cosh r_{1}x-a_{4}k_{2}\sinh r_{1}x \end{array}$$

where *a*_1_, *a*_2_, *a*_3_ and *a*_4_ are four parameters to be determined. The forces *F*_f_^e^ and *F*_p_^e^ in the FRP bar and the GFRP pipe, and the shear stress τ_fe_^e^ on the FRP bar-bonding layer interface and the relative slip δ^e^ between the FRP bar and the bonding layer are given below:

(30) $$\{\begin{array}{c} F_{f}^{e}=E_{f}A_{f}\left(a_{2}+a_{3}k_{1}r_{1}\sinh r_{1}x+a_{4}k_{1}r_{1}\cosh r_{1}x\right)\\ F_{p}^{e}=E_{p}A_{p}\left(a_{2}-a_{3}k_{2}r_{1}\sinh r_{1}x-a_{4}k_{2}r_{1}\cosh r_{1}x\right) \end{array}$$

(31) $$\tau_{\mathrm{fe}}^{e}=\frac{E_{f}A_{f}k_{1}r_{1}^{2}}{C_{f}}\left(a_{3}\cosh r_{1}x+a_{4}\sinh r_{1}x\right)$$

(32) $$\delta^{e}=\frac{E_{f}A_{f}k_{1}r_{1}^{2}\delta_{u}}{C_{f}\tau_{u}}\left(a_{3}\cosh r_{1}x+a_{4}\sinh r_{1}x\right)$$

(2) Softening part

In the softening part, with the combination of Equations (17) and (19), the relative displacement between the FRP bar and the GFRP pipe is:

(33) $$u_{f}^{s}-u_{p}^{s}-\frac{\delta_{r}\tau_{u}-\delta_{u}\tau_{r}}{\tau_{u}-\tau_{r}}=\left[\frac{r_{f}}{G_{e}}\ln(\frac{C_{p}}{C_{f}}\right)-\frac{\delta_{r}-\delta_{u}}{\tau_{u}-\tau_{r}}]\tau_{\mathrm{fe}}^{s}$$

In Equation (33), quantities with the superscript *s* are all defined in the softening part.

If *k*_3_, *k*_4_, and *r*_2_ are depicted with the following expressions:

(34) $$k_{3}=\frac{C_{f}}{E_{f}A_{f}\left[\frac{\delta_{r}-\delta_{u}}{\tau_{u}-\tau_{r}}-\frac{r_{f}}{G_{e}}\ln(\frac{C_{p}}{C_{f}}\right)]}$$

(35) $$k_{4}=\frac{C_{f}}{E_{p}A_{p}\left[\frac{\delta_{r}-\delta_{u}}{\tau_{u}-\tau_{r}}-\frac{r_{f}}{G_{e}}\ln(\frac{C_{p}}{C_{f}}\right)]}$$

(36) $$\delta_{0}=\frac{\delta_{r}\tau_{u}-\delta_{u}\tau_{r}}{\tau_{u}-\tau_{r}}$$

(37) $$r_{2}=\sqrt{k_{3}+k_{4}}$$

Then, Equations (6) and (12) can be simplified as:

(38) $$\{\begin{array}{c} \frac{d^{2}u_{f}^{s}}{dx^{2}}+k_{3}u_{f}^{s}-k_{3}u_{p}^{s}=k_{3}\delta_{0}\;\;\;\\ \frac{d^{2}u_{p}^{s}}{dx^{2}}-k_{4}u_{f}^{s}+k_{4}u_{p}^{s}=-k_{4}\delta_{0} \end{array}$$

With the method for ordinary differential equation solution, the solved result of Equation (35) is:

(39) $$\left\{\begin{array}{c} u_{f}^{s}\\ u_{p}^{s} \end{array}\right\}=\eta exp(\mu x)+\left\{\begin{array}{c} \delta_{0}\\ 0 \end{array}\right\}$$

where η is a vector. With the combination of Equations (36) and (35) and considering the non-zero solution of Equation (35), the characteristic equation can be derived:

(40) $$\left|\begin{array}{c} \mu^{2}+k_{3}\;\;\;\;\;-k_{3}\\ -k_{4}\;\;\;\;\;\mu^{2}+k_{4} \end{array}\right|=0$$

By solving Equation (39), the values of μ are:

(41) $$\mu_{5}=\mu_{6}=0;{\;\mu }_{7}=-\mu_{8}=r_{2}i$$

The calculated characteristic vectors are:

(42) $$\eta_{5}=\eta_{6}=\left\{\begin{array}{c} 1\\ 1 \end{array}\right\};\eta_{7}=\eta_{8}=\left\{\begin{array}{c} k_{3}\\ -k_{4} \end{array}\right\}$$

With the characteristic vectors, the solutions to Equation (35) are:

(43) $$\{\begin{array}{c} u_{f}^{s}=a_{5}+a_{6}x+a_{7}k_{3}\cos r_{2}x+a_{8}k_{3}\sin r_{2}x+\delta_{0}\\ u_{p}^{s}=a_{5}+a_{6}x-a_{7}k_{4}\cos r_{2}x-a_{8}k_{4}\sin r_{2}x\; \end{array}$$

where *a*_5_, *a*_6_, *a*_7_, and *a*_8_ are four parameters to be determined. The forces *F*_f_^s^ and *F*_p_^s^ in the FRP bar and the GFRP pipe, and the shear stress τ_fe_^s^ on the FRP bar-bonding layer interface and the relative slip δ^s^ between the FRP bar and the bonding layer are given below:

(44) $$\{\begin{array}{c} F_{f}^{s}=E_{f}A_{f}\left(a_{6}-a_{7}k_{3}r_{2}\sin r_{2}x+a_{8}k_{3}r_{2}\cos r_{2}x\right)\\ F_{p}^{s}=E_{p}A_{p}\left(a_{6}+a_{7}k_{4}r_{2}\sin r_{2}x-a_{8}k_{4}r_{2}\cos r_{2}x\right) \end{array}$$

(45) $$\tau_{\mathrm{fe}}^{s}=-\frac{E_{f}A_{f}k_{3}r_{2}^{2}}{C_{f}}\left(a_{7}\cos r_{2}x+a_{8}\sin r_{2}x\right)$$

(46) $$\delta^{s}=\delta_{0}+\frac{E_{f}A_{f}k_{3}r_{2}^{2}(\delta_{r}-\delta_{u})}{C_{f}\left(\tau_{u}-\tau_{r}\right)}\left(a_{7}\cos r_{2}x+a_{8}\sin r_{2}x\right)$$

(3) Frictional part

In the frictional part, τ_fe_^e^ equals to τ_r_, thus Equations (6) and (12) are simplified as:

(47) $$\{\begin{array}{c} \frac{d^{2}u_{f}^{f}}{dx^{2}}=\frac{C_{f}}{E_{f}A_{f}}\tau_{r}\;\;\;\;\\ \frac{d^{2}u_{p}^{f}}{dx^{2}}=-\frac{C_{f}}{E_{p}A_{p}}\tau_{r} \end{array}$$

In Equation (47), quantities with the superscript *f* are all defined in the frictional part.

Thus, the solutions of Equation (47) are:

(48) $$\{\begin{array}{c} u_{f}^{f}=a_{9}+a_{10}x+\frac{C_{f}\tau_{r}}{2E_{f}A_{f}}x^{2}\;\;\\ u_{p}^{f}=a_{11}+a_{12}x-\frac{C_{f}\tau_{r}}{2E_{p}A_{p}}x^{2} \end{array}$$

where *a*_9_, *a*_10_, *a*_11_, and *a*_12_ are four parameters to be determined. The forces *F*_f_^f^ and *F*_p_^f^ in the FRP bar and the GFRP pipe, and the shear stress τ_fe_^f^ on the FRP bar-bonding layer interface and the relative slip δ^f^ between the FRP bar and the bonding layer are given below:

(49) $$\{\begin{array}{c} F_{f}^{f}=a_{10}E_{f}A_{f}+C_{f}\tau_{r}x\\ F_{p}^{f}=a_{12}E_{p}A_{p}-C_{f}\tau_{r}x \end{array}$$

(50) $$\tau_{\mathrm{fe}}^{f}=\tau_{r}$$

(51) $$\delta^{f}=a_{9}-a_{11}+\left(a_{10}-a_{12}\right)x+\left(\frac{1}{E_{f}A_{f}}+\frac{1}{E_{p}A_{p}}\right)\frac{C_{f}\tau_{r}x^{2}}{2}-\frac{\tau_{r}r_{f}}{G_{e}}\ln(\frac{C_{p}}{C_{f}})$$

3. The critical FRP bar anchorage length calculation

**Figure 6:**
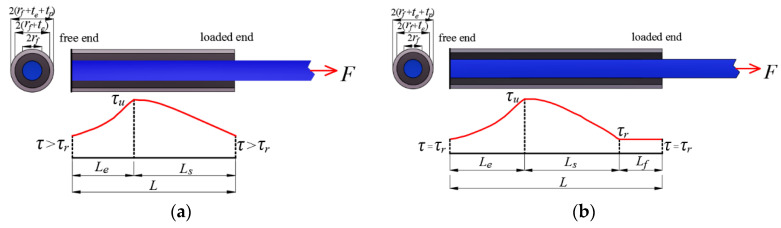
The shear stress envelop under the ultimate load transfer capacity:(**a**) for *L* < *L*_c_; (**b**) for *L* > *L*_c_.

**Figure 7:**
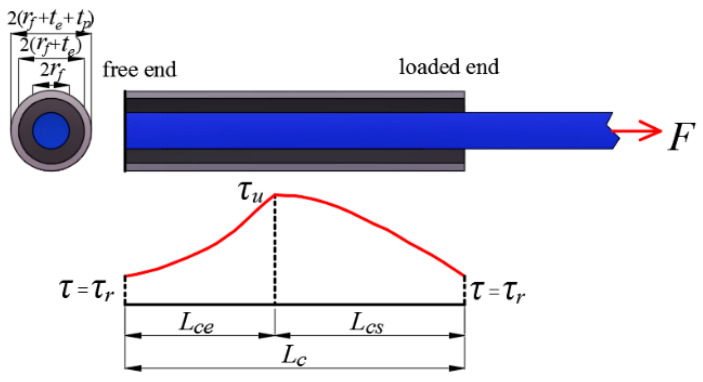
The shear stress envelop under the characteristic FRP bar anchorage length.

For the proposed resin-filled GFRP pipe connection system, the ultimate load transfer capacity of the FRP bar-bonding layer interface happens when the interfacial shear stress envelope is motivated to enclose the maximum area. However, considering the trilinear interfacial bond-slip curve, the shear stress envelop under the ultimate load transfer capacity may vary from two-part to three-part with the FRP bar anchorage length variation under the given sectional configuration (as seen in Figure 6). In view of this, a characteristic FRP bar anchorage length *L_c_* is introduced in the paper to characterize the threshold at which the shear stress envelop varies from two-part to three-part. With the definition of *L_c_*, it corresponds to the FRP bar anchorage length at which the shear stress distribution at the two ends (as seen in Figure 7) are both motivated to equal the residual frictional stress. Thus, the corresponding boundary conditions are:

(52) $$F_{f}^{e}\left(0\right)=0,\;F_{p}^{s}\left(L_{\mathrm{cs}}\right)=0,u_{p}^{e}\left(0\right)=0$$

(53) $$F_{f}^{e}\left(L_{\mathrm{ce}}\right)=F_{f}^{s}\left(0\right),\;F_{p}^{e}\left(L_{\mathrm{ce}}\right)=F_{p}^{s}\left(0\right),\;u_{f}^{e}\left(L_{\mathrm{ce}}\right)=u_{f}^{s}\left(0\right),\;u_{p}^{e}\left(L_{\mathrm{ce}}\right)=u_{p}^{s}\left(0\right)$$

(54) $$\delta^{e}\left(0\right)=\frac{\tau_{r}\delta_{u}}{\tau_{u}},\delta^{e}\left(L_{\mathrm{ce}}\right)=\delta_{u},\delta^{s}\left(L_{\mathrm{cs}}\right)=\delta_{r}$$

Solving Equations (52)–(54) simultaneously, the parameters *a*_1_–*a*_8_ and the corresponding lengths of the elastic part (*L*_ce_) and softening part (*L*_cs_) of the characteristic FRP bar anchorage length are both obtained. Thus, the characteristic FRP bar anchorage length (*L*_c_) and the corresponding ultimate load transfer capacity (*F*_c_) of the interface are given as:

(55) $$L_{c}=L_{\mathrm{ce}}+L_{\mathrm{cs}}$$

(56) $$F_{c}=2\pi r_{f}\left(\overset{L_{\mathrm{ce}}}{\underset{0}{\int }}\tau_{\mathrm{fe}}^{e}dx+\overset{L_{\mathrm{ce}}+L_{\mathrm{cs}}}{\underset{L_{\mathrm{ce}}}{\int }}\tau_{\mathrm{fe}}^{s}dx\right)$$

With the comparison between the ultimate load transfer capacity (*F*_c_) of the interface and the tensile capacity (*F*_fu_) of the spliced FRP bars, the critical FRP bar anchorage length (i.e., GFRP pipe length) in connection system design can be determined with the following three cases.

(1) Case 1: for *F*_fu_ < *F*_c_

In this case, a smaller FRP bar anchorage length is needed to provide a load transfer capacity that ensures FRP bar rupture and the shear stress envelop at FRP rupture is made up of an elastic part and a softening part and the boundary conditions are:

(57) $$F_{f}^{e}\left(0\right)=0,\;F_{f}^{s}\left(L_{s}\right)=F_{\mathrm{fu}},\;F_{p}^{s}\left(0\right)=F_{\mathrm{fu}},\;F_{p}^{s}\left(L_{s}\right)=0,\;u_{p}^{e}\left(0\right)=0$$

(58) $$F_{f}^{e}\left(L_{e}\right)=F_{f}^{s}\left(0\right),\;F_{p}^{e}\left(L_{e}\right)=F_{p}^{s}\left(0\right),\;u_{f}^{e}\left(L_{e}\right)=u_{f}^{s}\left(0\right),\;u_{p}^{e}\left(L_{e}\right)=u_{p}^{s}\left(0\right)$$

(59) $$\delta^{e}\left(L_{e}\right)=\delta_{u}$$

Solving Equations (57)–(59) simultaneously, the parameters *a*_1_–*a*_8_ and the corresponding lengths of the elastic part (*L*_e_) and softening part (*L*_s_) are both obtained and the critical FRP bar anchorage length *L* is equal to:

(60) $$L=L_{e}+L_{s}$$

(2) Case 2: for *F*_fu_ = *F*_c_

In this case, the critical FRP bar anchorage length *L* is equal to:

(61) $$L=L_{c}=L_{\mathrm{ce}}+L_{\mathrm{cs}}$$

(3) Case 3: for *F*_fu_ > *F*_c_

In this case, a larger FRP bar anchorage length is needed to provide a load transfer capacity that ensures FRP bar rupture and the shear stress envelop at FRP rupture is made up of an elastic part, a softening part, and a frictional part and the boundary conditions are:

(62) $$F_{f}^{e}\left(0\right)=0,\;F_{f}^{f}\left(L_{f}\right)=F_{\mathrm{fu}},\;F_{p}^{f}\left(L_{f}\right)=0,\;u_{p}^{e}\left(0\right)=0$$

(63) $$F_{f}^{e}\left(L_{e}\right)=F_{f}^{s}\left(0\right),\;F_{p}^{e}\left(L_{e}\right)=F_{p}^{s}\left(0\right),\;u_{f}^{e}\left(L_{e}\right)=u_{f}^{s}\left(0\right),\;u_{p}^{e}\left(L_{e}\right)=u_{p}^{s}\left(0\right)$$

(64) $$F_{f}^{s}\left(L_{s}\right)=F_{f}^{f}\left(0\right),\;F_{p}^{s}\left(L_{s}\right)=F_{p}^{f}\left(0\right),\;u_{f}^{s}\left(L_{s}\right)=u_{f}^{f}\left(0\right),\;u_{p}^{s}\left(L_{s}\right)=u_{p}^{f}\left(0\right)$$

(65) $$\delta^{e}\left(0\right)=\frac{\tau_{r}}{\tau_{u}}\delta_{u},\delta^{e}\left(L_{e}\right)=\delta_{u},\delta^{s}\left(L_{s}\right)=\delta_{r}$$

Solving Equations (62)–(65) simultaneously, the parameters *a*_1_–*a*_12_ and the corresponding lengths of the elastic part (*L*_e_), softening part (*L*_s_) and frictional part (*L*_f_) are obtained and the critical FRP bar anchorage length is equal to:

(66) $$L=L_{e}+L_{s}+L_{f}$$

4. Determination of the GFRP pipe length

In determining the GFRP pipe length (i.e., the total anchorage length of the two spliced FRP bars), the fabrication errors (such as the errors introduced in components production, specimen fabrication) induced interfacial shear performance reduction should be considered. In view of this, a correction factor is introduced in the paper to compensate for the reduced load transfer capacity of the interface by enlarging the FRP bar anchorage length (i.e., by increasing the bonding area). Thus, the GFRP pipe length can be generally expressed as:

(67) $$L_{p}=\varphi_{l}L_{l}+\varphi_{r}L_{r}$$

where, *L*_l_ and *L*_r_ are the theoretical FRP bar anchorage lengths calculated with the above-derived design equations for the left and the right FRP bars, respectively, as seen in Figure 3; φ_l_ and φ_r_ are the corresponding correction factors.

For the proposed connection system designed to connect FRP bars with the same diameter and material properties, the final GFRP pipe length (*L*_p_) can be simplified as:

(68) $$L_{p}=2\varphi_{l}L_{l}=2\varphi_{r}L_{r}$$

## 3. Experimental Verification

To evaluate the effectiveness of the proposed connection system and the derived design formulas, a resin-filled GFRP pipe connection system was designed for splicing 16 mm in diameter BFRP bars and tested under unidirectional tension to study the tensile performance.

### 3.1. Specimen Design

**Figure 8:**
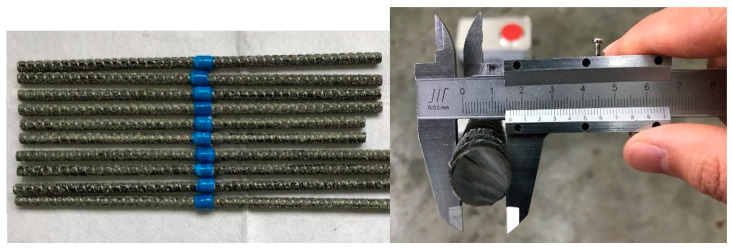
BFRP bars.

**Figure 9:**
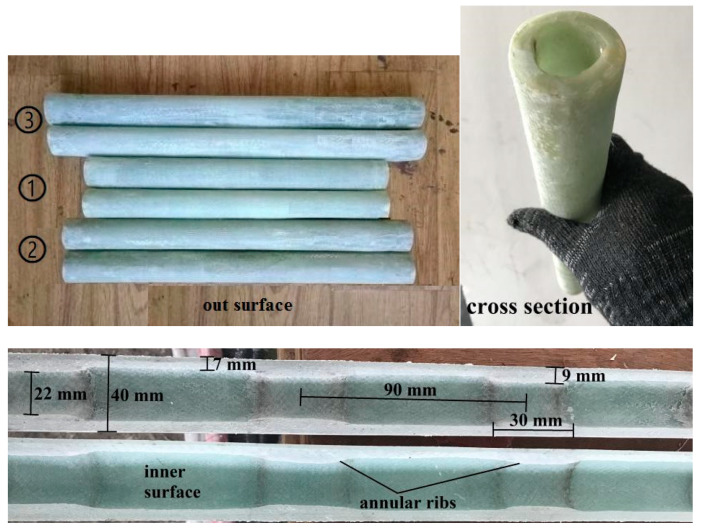
The designed GFRP pipe.

The BFRP bars spliced in the present study had a nominal diameter of 16 mm and were fabricated with a fiber volume ratio of 60%, as shown in Figure 8. According to the standard test method for mechanical properties of fiber-reinforced polymer bars [34], the measured average (±standard deviation) tensile strength of the BFRP bars was 930.2 ± 14.3 MPa. The GFRP pipes had a fiber volume ratio of 60% and the tested average tensile strength provided by the supplier was 420.7 ± 7.5 MPa from six specimens. To improve the mechanical interaction between the GFRP pipe and the grouted bonding layer, annular ribs with a height of 2 mm and length of 30 mm were designed along the inner surface of the GFRP pipe with a center-to-center space of 90 mm. With these parameters and Equation (1), the designed GFRP pipe had an inner diameter, an outer diameter, and a net pipe wall thickness of 26, 40, and 7 mm, respectively, as seen in Figure 9. The GFRP pipes were all fabricated with the vacuum assisted resin infusion molding (VARIM) technology.

The grout material used in the experiment was a two-component epoxy resin. According to the specifications from general testing methods for construction adhesives [35], the measured mechanical properties of the epoxy resin were all listed in Table 1. In the previous design of the GFRP pipe length, a series of standard interfacial shear tests were conducted to determine the BFRP bar-bonding layer interfacial shear behavior according to the specification of general testing methods for construction adhesives [35]. The tested critical values of the simplified tri-linear bond-slip curve are all listed in Table 2. Based on these parameters and the above-derived equations, the calculated characteristic anchorage length (*L*_c_) and the critical anchorage length (*L*) for the BFRP bars spliced in the present study were 265 and 230 mm, respectively.

**Figure 10:**
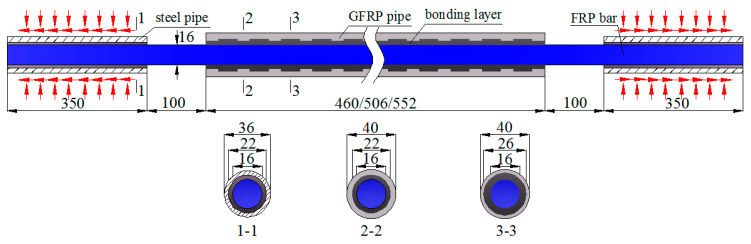
Specimen configurations (Unit in mm).

Based on the designed connection system, nine specimens were fabricated and tested under unidirectional tension to study their tensile performance. The nine specimens can be classified into three groups with the variation of the BFRP bar anchorage length. The designed anchorage lengths for the three groups were 230 mm (1.0*L*), 253 mm (1.1*L*), and 276 mm (1.2*L*), respectively, as listed in Table 3. Besides, to avoid direct clamping-induced premature failure of the BFRP bars, two clamping ends were formed for each specimen by inserting one end of the BFRP bars into resin-filled round steel pipes with a bonding layer thickness of 3 mm and anchorage length of 350 mm. The free part of the BFRP bars was designed to be 100 mm for all specimens, as seen in Figure 10.

### 3.2. Measuring Points and Loading Equipment

**Figure 11:**
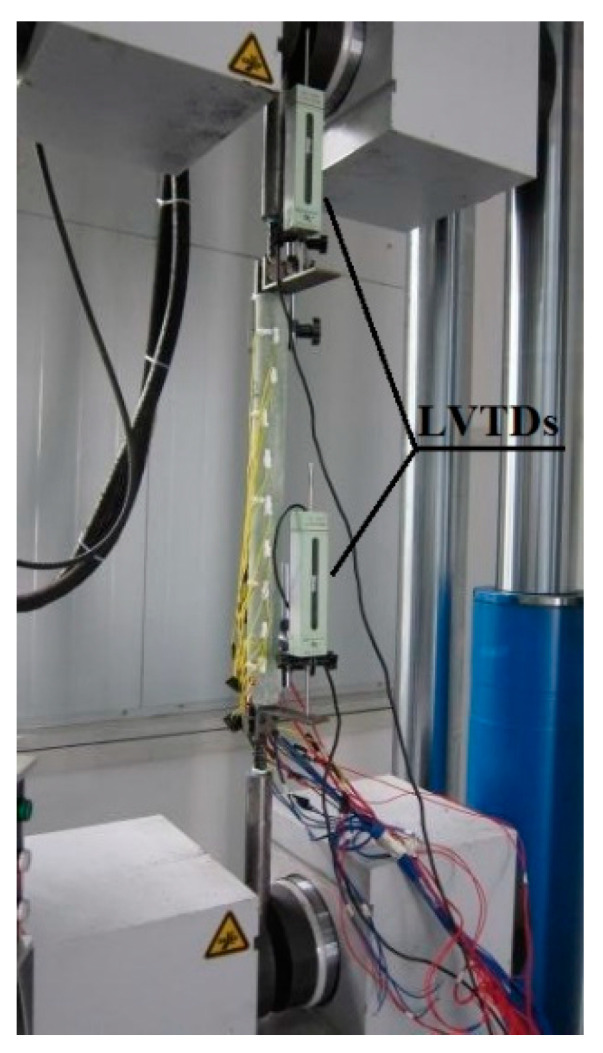
Loading equipment and measuring points.

Two LVDTs were arranged symmetrically at both ends of the GFRP pipe to measure the relative slips between the pipe and the BFRP bars. The tensile tests were conducted using a unidirectional tensile testing machine with a capacity of 600 kN. For each specimen, the two ends were clamped by the chunks of the testing machine and the load was applied at the rate of 0.2 mm/min, as seen in Figure 11. The relative slips were recorded by a computer-controlled data acquisition system with a rate of 5 Hz.

### 3.3. Test Results and Discussion

**Figure 12:**
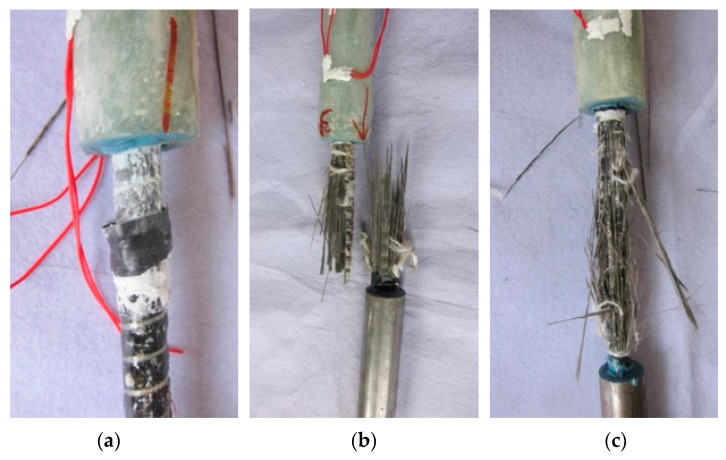
Typical tensile failure modes: (**a**) BFRP bar pullout (S-230-1); (**b**) BFRP bar rupture (S-253-1); and (**c)** BFRP bar rupture (S-276-1).

The observed failure modes of the tested specimens were BFRP bar pullout and BFRP bar rupture, as seen in Figure 12. For Group 1, the three specimens all failed in the BFRP bar pullout. Whereas, for specimens in Groups 2 and 3, the observed failure mode was BFRP bar rupture. The tested ultimate tensile capacities of all specimens and the corresponding relative slips, the theoretical ultimate load transfer capacities of the splices and the theoretical relative slips at BFRP bar rupture are all listed in Table 3. It should be mentioned that the tested relative slips presented in Table 3 were all recorded on LVDTs near the failure end.

The failure mode of BFRP bar pullout for specimens in Group 1 was mainly caused by the errors introduced in specimen fabrication, which significantly reduce the load transfer capacity and the shear stiffness of the BFRP bar-bonding layer interface. This can also be demonstrated by the measured lower ultimate tensile capacities and larger relative slips when compared to the corresponding theoretical values, as seen in Table 3.

With the increase of BFRP bar anchorage length, the fabrication error-induced interfacial mechanical reduction can be compensated to some extent, and thus the splices in Groups 2 and 3 provided the load transfer capacities beyond the tensile capacity of the spliced BFRP bars that ensures BFRP bar rupture. Whereas the fabrication error-induced interfacial shear stiffness reduction can also be observed for specimens in Groups 2 and 3 with the comparison between the theoretical values and the tested relative slips (as shown in Table 3).

**Figure 13:**
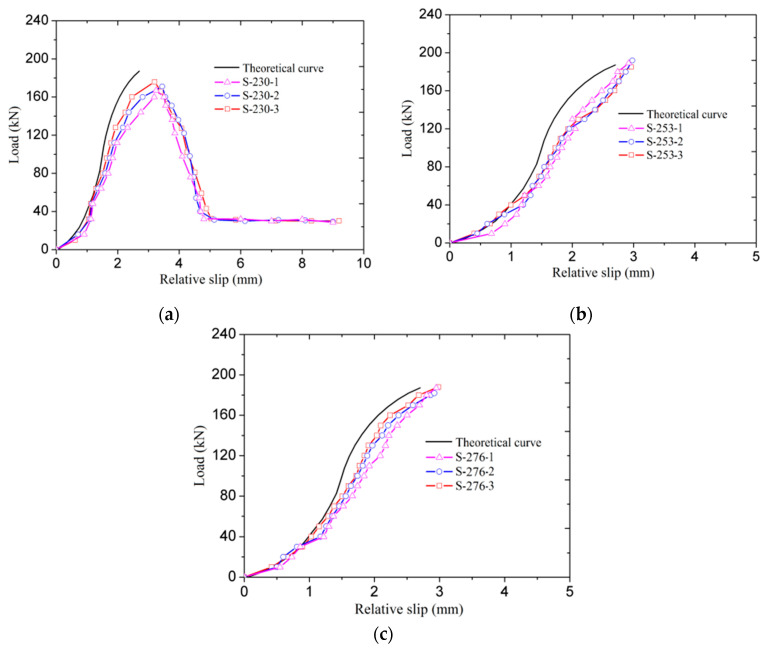
Experimental and theoretical load-slip curves of the specimens: (**a**) Group 1; (**b)** Group 2; and (**c**) Group 3.

Figure 13 shows the theoretically derived and the measured load-relative slip curves of all test specimens. The theoretical load-relative slip curves were calculated with the formulas derived above, and the measured load-relative slip curves were all recorded on LVDTs near the failure end. As can be observed from the figures, apart from Figure 13a, the measured load-relative slip curves are all matched well with the theoretically derived curves in the form of a constant increase in slips before the final failure occurred. As referring to specimens in Group 1, the external descending and horizontal parts are mainly caused by the premature failure mode of BFRP bar pullout. With this failure mode, the external softening and frictional parts were observed after the peak load. Besides, it should be mentioned that all specimens showed larger measured slips than the theoretical values under a given load. These behaviors are also caused by the fabrication error-induced mechanical reduction of the BFRP bar-bonding layer interface, which reduces the interfacial shear stiffness and the load transfer capacity.

With a deep comparison between the tested results and the theoretical results, although there are differences, they are matched well and thus the effectiveness of the proposed connection system and the derived design formulas are both verified. For the BFRP bars spliced in the present study, a minimum correction factor of 1.1 should be adopted in connection system design. Whereas it should be noted that designing the resin-filled GFRP pipe connection system for splicing FRP bars is beyond the scope of the present study, external experimental tests should be conducted to determine the correction factor for determining the final GFRP pipe length.

## 4. Conclusions

A new resin-filled GFRP pipe connection system was proposed for the butt splicing of FRP bars. With the proposed connection system and a simplified trilinear interfacial bond-slip model, a set of design formulas were derived based on the requirement that the proposed connection system should provide a load transfer capacity beyond the tensile capacity of the spliced FRP bars. At last, to evaluate the effectiveness of the proposed connection system and the derived design formulas, nine specimens were fabricated and tested under unidirectional tension to study their tensile performance. Based on the comparison between the theoretical and experimental results, the following conclusions can be drawn:

- Bond-type connection system is able to connect symmetrically placed FRP bars for splicing application. The proposed resin-filled GFRP pipe connection system can provide a sound connection and simple mechanism for the butt splicing of the BFRP bars studied herein.
- The derived design formulas can be used to determine the geometrical configuration of the proposed resin-filled GFRP pipe connection system for the given FRP bars. Besides, these formulas can also be reversely used to predict the load transfer performance of the splices with reasonable accuracy.
- Considering the fabrication error-induced load transfer capacity reduction of the FRP bar-bonding layer interface, a correction factor should be introduced in the connection system design to compensate for the reduced load transfer capacity of the interface by enlarging the FRP bar anchorage length. Thus, in designing the proposed connection system for the butt splicing of FRP bars, external experimental tests should be conducted to determine the reasonable correction factor for determining the final GFRP pipe length. For the BFRP bars studied in this paper, a minimum correction factor of 1.1 was found to ensure that the interface provides a load transfer capacity beyond the tensile capacity of the BFRP bars.
- Although the resin-filled GFRP pipe connection system showed good mechanical performance under unidirectional tension, the behaviors under reversal, dynamic, and sustained loads should be further studied before field application.

## Figures and Tables

**Table 1:** Material properties.

Material	Elastic Modulus /GPa	Ultimate Tensile Strength /MPa	Compressive Strength /MPa	Shear Strength /MPa	Poisson’s Ratio	Elongation
GFRP pipe	26.4 ± 0.5	420.7 ± 7.5	-	-	0.31	1.61 ± 0.05%
BFRP bar	54.1 ± 0.8	930.2 ± 14.3	-	-	0.29	1.72 ± 0.05%
Epoxy resin	2.3 ± 0.1	63.6 ± 2.7	124.5 ± 3.8	35.3 ± 2.1	0.38	2.75 ± 0.07%

Note: the elastic modulus, the ultimate tensile strength, the compressive strength, and the shear strength listed in Table 1 were all calculated using the nominal cross-sectional area of the specimens.

**Table 2:** Parameters of the bond-slip model.

Epoxy Resin	τ_u_/MPa	τ_r_/MPa	δ_u_/mm	δ_r_/mm
JGN	25.7 ± 0.6	2.8 ± 0.2	1.4 ± 0.2	3.2 ± 0.3

**Table 3:** Specimen details and the corresponding tested results.

Group Number	Specimen ID	BFRP Bar Anchorage Length/mm	GFRP Pipe Length/mm	Tested Ultimate Tensile Capacity/kN	Theoretical Ultimate Load Transfer Capacity/kN	Tested Relative Slip at Failure/mm	Theoretical Relative Slip at Failure/mm	Failure Mode
Group 1	S-230-1	230	460	176.3	187.4	3.20	3.10	BFRP bar Pullout
	S-230-2			171.5		3.45		
	S-230-3			167.9		3.25		
Group 2	S-253-1	253	506	185.4	201.7	2.96	2.91	BFRP bar rupture
	S-253-2			192.1		2.98		
	S-253-3			189.5		2.92		
Group 3	S-276-1	276	552	188.3	208.6	2.88	2.82	BFRP bar rupture
	S-276-2			182.2		2.92		
	S-276-3			187.6		2.95		

## Data Availability

The data presented in this study are available on request from the corresponding author. The data are not publicly available due to privacy.
